# Late relapses in leprosy patients in Brazil: 10-year post-trial of uniform multidrug therapy (U-MDT/CT-BR)

**DOI:** 10.1016/j.bjid.2024.103745

**Published:** 2024-04-30

**Authors:** Gerson Oliveira Penna, Maria Araci de Andrade Pontes, Sinésio Talhari, Heitor de Sá Gonçalves, Carolina Talhari, Allen de Souza Pessoa, Valderiza Pedroza, Samira Bührer-Sékula, Mariane Martins de Araujo Stefani, Maria Lucia Fernandes Penna

**Affiliations:** a Universidade de Brasília, Núcleo de Medicina Tropical, Brasília, DF, Brazil; b Fundação Oswaldo Cruz, Escola de Governo Fiocruz Brasília, Brasília, DF, Brazil; c Centro de Referência em Dermatologia Dona Libânia, Fortaleza, CE, Brazil; d Fundação Hospitalar de Dermatologia Tropical e Venereologia Alfredo da Matta, Manaus, AM, Brazil; e Universidade do Estado do Amazonas, Programa de Pós-Graduação em Ciências Aplicadas à Dermatologia, Manaus, AM, Brazil; f Universidade Federal de Goiás, Instituto de Patologia Tropical e Saúde Pública, Goiânia, GO, Brazil; g Universidade Federal Fluminense, Departamento de Epidemiologia e Bioestatística, Rio de Janeiro, RJ, Brazil

**Keywords:** Leprosy, Relapse, Clinical trial, Uniform multidrug therapy

## Abstract

**Background:**

Leprosy is a neglected dermato-neurologic, infectious disease caused by *Mycobacterium leprae* or *M. lepromatosis*. Leprosy is treatable and curable by multidrug therapy/MDT, consisting of 12 months rifampicin, dapsone and clofazimine for multibacillary/MB patients and for 6 months for paucibacillary/PB patients. The relapse rate is considered a crucial treatment outcome. A randomized Controlled Clinical Trial (U-MDT/CT-BR) conducted from 2007‒2012 compared clinical outcomes in MB patients after 12 months regular MDT/R-MDT and 6 months uniform MDT/U-MDT in two highly endemic Brazilian areas.

**Objectives:**

To estimate the 10 years relapse rate of MB patients treated with 6 months U-MDT.

**Methods:**

The statistical analyses treated the data as a case-control study, sampled from the cohort generated for the randomized trial. Analyses estimated univariate odds ratio and applied logistic regression for multivariate analysis, controlling the confounding variables.

**Results:**

The overall relapse rate was 4.08 %: 4.95 % (16 out of 323) in the U-MDT group and 3.10 % (9 out of 290) in the regular/R-MDT group. The difference in relapse proportion between U-MDT and R-MDT groups was 1.85 %, not statistically significant (Odds Ratio = 1.63, 95 % CI 0.71 to 3.74). However, misdiagnosis of relapses, may have introduced bias, underestimating the force of the association represented by the odds ratio.

**Conclusions:**

The relapse estimate of 10 years follow-up study of the first randomized, controlled study on U-MDT/CT-BR was similar to the R-MDT group, supporting strong evidence that 6 months U-MDT for MB patients is an acceptable option to be adopted by leprosy endemic countries worldwide.

**Trial registration:**

ClinicalTrials.gov: NCT00669643.

## Introduction

*Mycobacterium leprae* is a highly infectious microorganism with low virulence, resulting in only a small proportion of infected individuals manifesting disease. The clinical manifestations of leprosy encompass a broad spectrum of dermato-neurologic manifestations, reflecting the interaction between the bacilli and the host's immune response.^1^ Leprosy control programs primarily rely on early diagnosis and treatment, aiming to eliminate infectious sources and to interrupt *M. leprae* transmission chain. In 1997, the World Health Organization (WHO) proposed an operational classification system based on the number of skin lesions as a proxy for bacteriological load. Two Multidrug Therapy (MDT) regimens for leprosy were proposed: twelve months of daily dapsone plus clofazimine and monthly rifampicin doses for multibacillary/MB patients (> 5 skin lesions),^2^ while daily dapsone and monthly rifampicin doses^3^, ^4^, ^5^ regimen was employed for paucibacillary/PB patients (≤5 skin lesions). After the WHO recommendation in 2021, Brazil officially adopted a unified treatment with dapsone, clofazimine and rifampicin to all leprosy cases, regardless of being classified as a MB or a PB patient, however lasting six months for PB patients and 12 months for MB patients. In leprosy, the relapse rate has been considered a crucial treatment outcome.^6^ The duration of treatment for leprosy and tuberculosis has always been a controversial topic.^6^

The multidrug therapy for leprosy recommended by the WHO reduced the duration of treatment, resulting in a decline of disease prevalence, however without an impact on incidence, as many countries continue to report high detection rates. Globally the COVID-19 pandemic caused an important impact on the notifications of new leprosy cases and on the detection rate, with a significant reduction from 202.475 cases in 2019 to 140.594 cases in 2021. Compared to 2021 data, in 2022 174.087 new leprosy cases were reported representing an increase of 23.8%, still around 15% lower than the total rate recorded in 2019.^7^^,^^8^

Since the implementation of MDT in the early 1980s, no new standard treatment scheme has been proposed for leprosy. In many endemic countries, leprosy remains an uncontrolled infectious disease and the effectiveness of diagnosis, treatment, and control programs remains challenging.^9^

In theory the duration of treatment plays a pivotal role in preventing relapse in leprosy and the use of three antibiotic drugs is important to prevent the selection of resistant bacilli.^10^

A randomized Controlled Clinical Trial (U-MDT/CT-BR) was conducted from 2007‒2012 in two highly endemic Brazilian areas to compare clinical outcomes in MB patients after 12 months regular MDT/R-MDT and 6 months uniform MDT/U-MDT.^11^ The aim of the current study was to analyse the relapse rates of MB leprosy patients, 10 years after the completion of the uniform 6 months drug regimen (U-MDT/CT-BR)^11^ compared to the regular 12 months MDT regimen. This study was based on well-documented medical registers of the U-MDT/CT-BR trial's participants in two highly endemic leprosy settings in Brazil.

## Methods

The complete methods, details and results of the U-MDT/CT-BR randomized Controlled Clinical Trial were described previously^11^ and in this study only pertinent information is reported. In brief, the U-MDT/CT-BR trial was conducted in two healthcare units, designed by the Brazilian Ministry of Health as National Reference Centres for Leprosy: Dona Libânia Dermatology Centre, located in Fortaleza, Ceará state, Northeast Brazil and Alfredo da Matta Foundation located in Manaus, Amazonas state, North Brazil. Both centres are responsible to treat all relapse cases in each town, as well as complex cases referred by the local general physicians in primary health units. This attribute allowed us the strategy to link the reported relapse cases to the 613 leprosy patients originally enrolled in the U-MDT/CT-BR Controlled Clinical Trial, since trial's participants also had a general registration file in each unity, in which the participation in the trial was flagged. Initially, a search and analysis of SINAN (National System of Notifiable Diseases) data was carried out to identify all the relapses that had occurred in the two participating centers between 2017 and 2022, in which a total of 393 leprosy relapses were identified. Among these, 25 patients were identified as participants of the U-MDT/CT-BR trial. The clinical and laboratory data of these patients were obtained from the individual Case Report Forms (CRFs) and from the medical records and compared with the data from study patients who did not relapse.

The statistical analyses treated the data as a case-control study sampled from the cohort generated in the randomized trial, estimating univariate odds ratio, and applying logistic regression for multivariate analysis, controlling the confounding variables.

The definition of relapses (CASES) was MB leprosy patients enrolled at U-MDT/CT-BR trial that attended any of the two recruiting centres after the completion of MDT due to the reappearance of signs and symptoms, not related to leprosy reactions, and/or symptomatic patients that had an increase in the Bacillary Index (BI), compared to the last BI reported after treatment completion. CONTROLS were defined as: MB leprosy patients enrolled at U-MDT/CT-BR trial who did not attend any of the enrolling centres and those who were assisted for causes other than relapses.

Originally, to evaluate the Bacilloscopic Index (BI) trend over time, from 180 days after the onset of treatment to the end of the follow-up of the clinical trial (5 years), we have previously fixed a multilevel linear model with mixed effects, i.e., a random intercept model.^11^ According to this model, the average BI (aBI) was the independent variable, and the dependent variables were time (in days), initial aBI (iBI) continuous and categorized as high (iBI ≥ 4) and low (iBI < 4), study arm (U-MDT = 1 and R-MDT = 0), relapse (present or not) and three interaction variables combining BI level, study arm and relapse time (days). For this analysis, time zero was considered from 180 days after the onset of treatment i.e., the time in which MB patients were randomized into R-MDT (12 months) or U-MDT (6 months) study arms. For clarity, the categorized initial BI/iBI is referred as iniBILevel (≥ 4 or < 4), in contrast with initial BI (iBI) and average BI (aBI) which refer to continuous measure of initial and follow-up BI, the average BI of all sites of smear collection. For clarity, as for each patient, multiple BI measures were taken during the study and as each BI measure is composed of different BIs in different body areas/collection sites, the categorized initial BI/iBI is referred as iniBILevel (≥ 4 or < 4), whereas initial BI (iBI) refers to the measure of initial BI in all sites and the average BI (aBI) refers to the average BI in all sites of smear collection.

## Ethics considerations

This study was performed under the international (Helsinki) and Brazilian research regulations and was approved by the National Ethics Commission of Research (CONEP) of the Ministry of Health, protocol number 12949/2007. Written informed consent was required from all the patients prior to their inclusion in the study. For patients aged six to 17 years, written parental consent was mandatory. Data confidentiality was strictly guaranteed. Patients were free to leave the study, if they desired, and opt for the R-MDT regimen outside the study.

## Results

The recruiting phase of the U-MDT/CT-BR trial took place from 2007 to 2012, and each patient was actively followed up for 5 years. In 2017, an official trial publication reported four relapse cases.^11^ In the current 10 years U-MDT follow-up study, 21 additional relapse cases linked to the trial are reported. Therefore, this study describes the association of the total number of relapses (21 new cases identified, plus the 4 cases already reported)^11^ with other variables, and the comparison of relapse rates in the U-MDT (6 doses) and the R-MDT (12 doses) regimens.

The total number of 25 relapse cases identified amongst the original 613 MB leprosy patients who were randomly assigned to different study arms (U-MDT and R-MDT) in the U-MDT/CT-BR trial, represents an overall relapse proportion of 4.08 %. According to this data, the separate analysis of the study arms showed that the relapse proportion was 4.95 % (16 out of 323) in the U-MDT group and 3.10 % (9 out of 290) in the R-MDT group. The difference in relapse proportion between U-MDT and R-MDT study arms was < 2 % (1.85 %) and did not reach statistical significance (Odds Ratio = 1.63, 95 % CI 0.71 to 3.74).

**Fig. 1:**
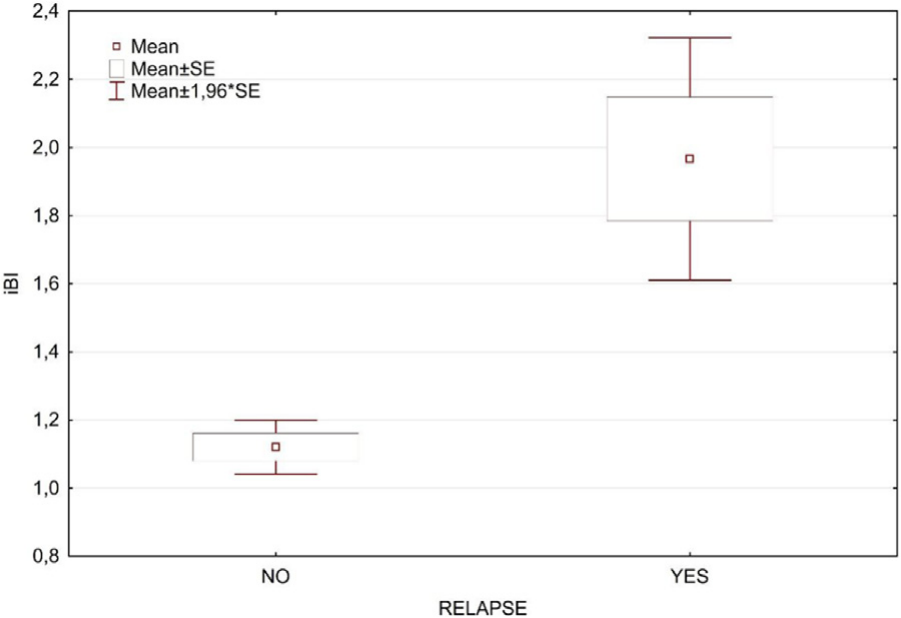
Box plot distributions of the initial BI (iBI) in the two groups defined by the occurrence of leprosy relapse.

Table 1 presents a breakdown analysis of leprosy relapse cases categorized by gender and initial BI level (≥ 4, < 4). The occurrence of leprosy relapse was not associated with initial BI level nor gender. Table 2 displays the *t*-test results for the initial BI (iBI) and age, categorized according to the occurrence of relapse, with a very small and significant p-value for iBI, while age was not associated to relapse. In the univariate analyses, the only statistically significant variable was related to the initial BI (Fig. 1).

**Table 1:** Distribution of relapses by initial BI level and sex.

		**Mean no relapse group**	**Mean relapse group**	**Odds Ratio**	**95 % IC**
**IniBILevel**	< 4	306	8	2.30	0.97:5.42
	Column%	52.04 %	32.00 %		
	≥ 4	282	17		
	Column%	47.96 %	68.00 %		
	Totals	588	25		
**Gender**	Male	395	15	1.36	0.60:3.09
	Column%	67.18 %	60.00 %		
	Female	193	10		
	Column%	32.82 %	40.00 %		
	Totals	588	25		

IniBILevel, initial bacilloscopic index level categorized as ≥ 4 and < 4.

**Table 2:** Initial BI and age *t*-test results for continuous variables according to relapse occurrence.

**Variable**	**Mean relapse group**	**Mean no relapse group**	**t-value**	**df**	**p**
iBI	3.62	2.43	2.96	611	0.0000
Age	40.45	35.19	1.78	611	0.08

iBI, Initial Bacilloscopic Index; df, Degree of Freedom.

To mitigate the potential impact of confounding variables and to ensure the accuracy of the findings, we performed a logistic regression analysis with relapse as the dependent variable. The independent variables considered in the regression model were gender, age, initial BI, initial BI level, and treatment groups (R-MDT and U-MDT). This comprehensive approach enabled us to estimate the Odds Ratios for relapse while effectively controlling for the influence of other variables. The results of the logistic regression analysis show that the odds of leprosy relapse in the U-MDT and the R-MDT treatment groups observed in the univariate analysis (Odds = 1.63, data not shown) is close to the odds estimated by the multivariable analyses (Odds = 1.7), (Table 3) indicating a small influence of confounding variables.

**Table 3:** Odds Ratio of relapse estimated by logistic multivariable regression.

	**Odds Ratio**	**Lower CL 95.0 %**	**Upper CL 95.0 %**	**p-value**
**Ibi**	2.11	1.28	3.49	0.00
**Age**	0.98	0.95	1.00	0.08
**Sex**	0.50	0.21	1.18	0.12
**iniBILevel**	0.19	0.03	1.19	0.08
**Study arms**	1.70	0.73	3.97	0.22

iBI, Inititial Bacilloscopic Index; iniBILevel, Initial Bacilloscopic Index Level; Study arms, U-MDT; R-MDT; CL, Confidence Limit.

The mean time of relapse since the first visit was 9.8 years (median 9.40 years) in the 6-month U-MDT and 9.91 years (median 9.7 years) in the 12-month R-MDT group and 68 % of all relapses were registered before 10 years of follow-up.

**Fig. 2:**
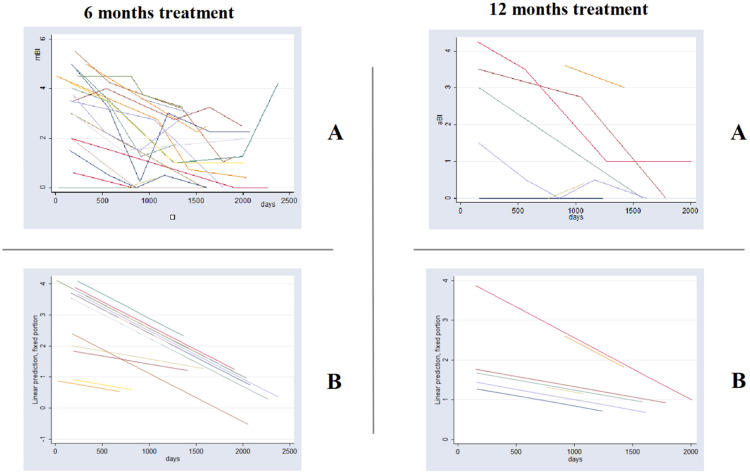
Graph of average Bacilloscopic Index (aBI) vs. time in each treatment group (6 months U-MDT and 12 months R-MDT) of patients with relapse: (A) As was observed and (B) The predicted values by statistical model presented in Table 4.

Fig. 2 shows among relapse cases, the linear adjusted average Bacilloscopic Index (aBI) as a function of time. This figure illustrates the need for a multilevel model for analysis, as we are dealing with multiple BI measures (average BI/aBI) of the same patient overtime. This analysis approach considers the aBI time trend of each patient instead of considering the aBI of all patients in each time point, according to treatment duration. The full mixed effects model adjusted for the aBI trend considered the following independent variables: treatment group, aBI level, initial aBI, relapse and time, plus three interaction variables ‒ time and relapse; time and group; iBI level and time.

This analysis among leprosy relapse cases showed no statistical significance for the regression coefficient of the bacilloscopic index of treatment groups U-MDT compared to R-MDT and for interaction variables that included treatment group (“group X time” and “group X initial aBI”). The full model among relapses allowed us to estimate treatment effect on aBI value, on time trend of aBI value and on different effect according to initial aBI (Table 4). The likelihood ratio test of this model and simple linear regression showed a p-value < 0.00001 with a higher likelihood for the multilevel model.

**Table 4:** Relapse cases: multilevel linear model with mixed effects of bacilloscopic index decrease ‒ fixed parameters.

**aBI**	**Coef.**	**Std. Err.**	**z**	***p*>*z***	**[95 % Conf.**	**Interval]**
days	−0.0006	0.0001	−7.8700	0.0000	−0.0007	−0.0004
iBILevel	1.6314	0.1337	12.2100	0.0000	1.3694	1.8933
Relapse	0.5556	0.2210	2.5100	0.0120	0.1225	0.9887
Study arm	−0.0337	0.0950	−0.3500	0.7230	−0.2198	0.1524
iBI	0.3232	0.0299	10.8000	0.0000	0.2646	0.3819
Relapse*days	0.0001	0.0002	0.5500	0.5810	−0.0002	0.0004
iBILevel*days	−0.0010	0.0001	−12.9300	0.0000	−0.0012	−0.0009
Study arm*days	0.0001	0.0001	0.7800	0.4370	−0.0001	0.0002
_cons	0.2655	0.0911	2.9100	0.0040	0.0869	0.4441

Coef., Coefficient; Std. Err., Standard Error; z, Z-score; *P* > *z*, p-value for the Z-score; [95 % Conf. Interval], 95 % Confidence Interval.

## Discussion

For leprosy treatment, the relapse rate has always been considered a crucial MDT outcome measure.^6^ This is a 10-year follow-up study of MB patients after a randomized Controlled Clinical Trial (U-MDT/CT-BR) of 6 months uniform MDT which was compared to 12 months regular MDT leprosy treatment. The 10 years follow-up data of MB patients showed that the difference in the relapse frequency between the U-MDT and R-MDT groups was 1.8 % (*p* > 0.05). It is important to highlight that the most important point of our study is the lack of significant statistical differences in relapse rates between the two treatment groups, indicating no programmatic relevance for leprosy control programs.

From 2017 to 2022 the two recruiting centers, which are in charge of the diagnosis and management of overall local leprosy relapses, reported a total of 393 relapses among MB patients. From these, 25 relapses were diagnosed in patients that have participated in the U-MDT/CT-BR trial, while the remaining 368 relapses referred to patients outside the trial that received the regular MDT (at least 12 doses for MB leprosy). The current study also showed that among U-MDT/CT-BR trial participants the occurrence of leprosy relapse was not associated with gender, nor age, nor treatment duration (6 or 12 months). The analyses performed showed that the only statistically significant association observed was between leprosy relapse rate and the average Bacilloscopic Index (aBI), confirming the prognostic importance of the BI in leprosy.

The analysis of the average BI (aBI) data trend was performed using the same data set employed for the 2017 publication, i.e., data from the trial period (5 years after treatment completion), with the inclusion of relapse as a covariate.^11^ On the regression model of aBI trend, the coefficient of relapse was significant, pointing out that the aBI trend in an earlier period has prognostic value for future occurrence of relapses. One possible interpretation of this result is that this association of relapse and aBI trend may be related to low compliance with longer treatment regimens, however this hypothesis deserves further investigation using a larger data bank.

Our analyses showed that the mean time of relapse occurrence in MB patients was less than 10 years since the first visit: 9.8 years in the 6-month treatment (U-MDT) and 9.91 years in the 12-month treatment group (R-MDT). In fact, almost 70% of all relapses in MB patients were registered before 10 years of follow-up. A study conducted in the Philippines showed the results of relapses after 24 months treatment reporting a cumulative risk of relapse of 6.6%, and a mean time of occurrence of 10.5 years after the cure release.^16^ The current study showed a smaller relapse proportion, suggesting that the compliance to treatment seems to be lower when the proposed treatment is too long.

It should be pointed out that, despite the differences in the duration of treatment, relapses were associated with higher initial BI (iBI), indicating that high BI patients at diagnosis are indeed the ones that need to be closely monitored for the occurrence of relapses. However, the great majority of the 6-month treatment MB patients did not relapse during the 10 years period.

The U-MDT/CT-BR has recruited 613 MB patients and the final results were based on the analysis of all 439 MB patients that complied to the five years follow up period.^11^ In the current manuscript with 10 years follow up data, the multivariate analysis of Bilevel ≥ 4 and < 4 was not significant, and the impact of BI on relapses is indicated in Fig. 1.

Our study shows that in the 10 years follow-up, the overall relapse rate among MB patients of the U-MDT/CT-BR trial was 4.08%. During the peak of MDT campaigns, the WHO reported relapse rates to range between 6.5 and 30 per 1000 person-years for PB leprosy and 80 per 1000 person-years for MB leprosy.^12^ These data indicate the large variation of relapse rates reported in leprosy literature. The incidence rate of relapse is difficult to interpret, as it does not discriminate different risk according to the time since treatment completion. An incidence rate of relapses of 80 per 1000 person-years (or 8 per 100 py) as reported^12^ is equal to an absolute risk of 55% in 10 years. Comparisons of leprosy relapse rates with other studies are also problematic because of the different definitions of relapse adopted, as in some studies, leprosy reactions were included among relapse cases.^13^ As detailed in methods, in our study, all leprosy reactions were excluded from the relapse definition.

Our study estimated the relapse rate among MB patients that participated in the U-MDT/CT-BR trial. A recent leprosy study in Brazil showed a relapse risk of 11.9%, but the denominator of the risk was the number of cases reported in the same period as the relapses.^14^ However, the real population of treated leprosy cases that were at risk of relapse is not this one, an assumption that introduced a real bias. Considering this measure, i.e., the proportion of relapses among cases beginning leprosy treatment in a period, may be unreliable because the proportion of relapses may change according to the variation of the numerator or the denominator, therefore this calculation does not represent an epidemiologic measure. In this context, the temporal trend of the relapse ratio does not reflect the relapse risk temporal trend.^15^

While the overall relapse rate among MB patients of the U-MDT/CT-BR was 4.08% in 10 years follow-up, a Brazilian study estimated the relapse incidence density in a cohort of MB patients (bacilloscopic index/BI >0), diagnosed between September 1997 and June 2017, and treated with twelve doses of MB-MDT. Ten relapse cases were reported in a cohort of 713 patients followed-up for an average of 12.1 years, showing an incidence rate of 1.16 relapse cases per 1000 person-years and the cumulative risk was 2.5% in 20 years.^17^ This study among a similar number of MB patients described less than half the number of relapses as observed in our study. The possible reasons for this difference may be related to the type of follow-up (active × passive) and to different criteria adopted to define a relapse case. On the other hand, note that the relapse rate of 4.08% described here was slightly higher than reported in other studies and this could be related to the adoption of BI as an additional criterion for relapse, as it is known that BI takes many years to fall. Thus, if our study had not included the increase in BI as a criterion for relapse, it is possible that the real number of relapses could have been fewer than reported.^18^

The first three relapse cases observed among participants of U-MDT/CT-BR trial were analysed by *M. leprae* whole genome sequencing and by the identification of single nucleotide polymorphisms/SNPs in strains’ sequences obtained from paired biopsies of the skin lesions observed in the first and second disease episode.^19^ The SNPs analyses showed that in one case, instead of relapse, there was clear evidence of reinfection with an unrelated *M. leprae* strain, identified by different set of SNPs identified. In the other two cases described, according to the SNPs analyses, relapse of the original infection appeared more probable.^19^ In this sequencing study, no mutation responsible for resistance to rifampicin, dapsone or ofloxacin was found, therefore drug resistance was excluded as a possible cause of disease recurrence. Relapses are usually due to the growing of persistent bacilli, not to resistance to the MDT drugs. Since on the molecular level, the distinction of reinfection and relapse depends on whole genome *M. leprae* sequencing and analyses, we cannot exclude the possibility that in leprosy hyper endemic areas, a yet unknown proportion of reported cases of relapses may indeed correspond to cases of reinfection.

We acknowledge that the main limitation of our study was not estimating the real relapse risk or rate, once it is not possible to confirm that patients that were not diagnosed as relapses are indeed cured and alive. However, treating data as case-control allowed us to calculate the Odds Ratio that estimate the relative risk. Another potential limitation to be considered is the misdiagnosis of relapses, by the inclusion of leprosy reactions as relapses (cases) by physicians in charge of patients’ management after the trial conclusion. This may have introduced a small bias (due to few cases misclassified in the total number of no relapse cases), underestimating force the association, represented by the odds ratio. It is also important to acknowledge that some patients that were enrolled in the trial may have moved from their original living cities, so that we may have missed relapses detected in other health centers, but this risk should be equal to both study arms. Also, including other possible cases of relapses that moved away would not allow us to maintain a standardized criteria for the definition of relapses, in order to avoid misdiagnosis or over diagnosis.

The relevance of our findings to leprosy control programs is the demonstration that unifying the duration of MDT treatment to all leprosy patients (MB and PB) to six months was not associated with harm or failure of treatment for MB disease, as no statistically significant difference of relapse proportion was observed when relapses rates were compared to the 12-month R-MDT. In other words, our results of 10-years follow-up of MB patients under 6-months and 12-months MDT showed that, there was no association between relapse rates and treatment duration. It should be highlighted that the shortened treatment strategy for MB patients to 6-months can also promote an important decrease of treatment adverse effects and reduce the number of follow-up visits to half. A lower patient load at the primary health unity can improve the relationship of the patient with the health service and increase compliance to treatment, resulting in a reduction of relapses, as low treatment compliance is strongly associated to relapse.^20^, ^21^, ^22^, ^23^, ^24^ Results from studies performed in Bangladesh, using the same period of follow up as used in our study, showed concordant results.^25^ This study performed in Brazil, together with results of studies performed China, India and Bangladesh corroborate our findings.^11^^,^^23^, ^24^, ^25^, ^26^

## Conclusion

The reported relapse rate of 10 years follow-up study of MB leprosy patients that participated in the first randomized and Controlled Clinical Trial U-MDT/CT-BR strongly supports the evidence that 6 months treatment with U-MDT for MB patients can be adopted as a shorter regimen promoting greater adherence, with a consequent lower rate of treatment abandonment, less chance of developing antimicrobial resistance and fewer adverse effects. Thus, the U-MDT, with 6 instead of 12 doses of treatment, can have wide applicability in a disease with a limited therapeutic arsenal, which is still a public health problem in leprosy endemic countries, including Brazil.

## Funding

U-MDT/CT-BR Leprosy Controlled Clinical Trial ± Brazil was funded by the Department of Science and Technology (DECIT) of Brazilian Ministry of Health and the Brazilian Council for Research (CNPq process #403293/2005-7). MMAS is a PVN-II Research Fellow from FAPEAM/Amazonas (“Programa de Apoio à Formação em Ciências Dermatológicas – PRODERM-RH, FUHAM, Manaus, Amazonas grant #010/2023). The funders had no role in study design, data collection and analysis, decision to publish, or preparation of the manuscript. The authors themselves received no specific funding for this work.

## Authors’ contributions

Gerson Oliveira Penna: Study concept and design; data collection, analysis and interpretation of data; statistical analysis; writing of the manuscript; data collection, analysis and interpretation; effective participation in the research guidance; intellectual participation in the propaedeutic and/or therapeutic conduct of the studied cases; critical review of the literature; final approval of the final version of the manuscript.

Maria Araci de Andrade Pontes: Study concept and design; data collection, analysis and interpretation of data; statistical analysis; writing of the manuscript; data collection, analysis and interpretation; effective participation in the research guidance; intellectual participation in the propaedeutic and/or therapeutic conduct of the studied cases; critical review of the literature; final approval of the final version of the manuscript.

Sinésio Talhari: Study concept and design; writing of the manuscript; effective participation in the research guidance; intellectual participation in the propaedeutic and/or therapeutic conduct of the studied cases; final approval of the final version of the manuscript.

Heitor de Sá Gonçalves: Study concept and design; writing of the manuscript; effective participation in the research guidance; intellectual participation in the propaedeutic and/or therapeutic conduct of the studied cases; final approval of the final version of the manuscript.

Carolina Talhari: Study concept and design; data collection, analysis and interpretation of data; data collection, analysis and interpretation; effective participation in the research guidance; final approval of the final version of the manuscript.

Allen de Souza Pessoa: Study concept and design; data collection, analysis and interpretation of data; data collection, analysis and interpretation; effective participation in the research guidance; final approval of the final version of the manuscript.

Valderiza Pedroza: Study concept and design; data collection, analysis and interpretation of data; data collection, analysis and interpretation; effective participation in the research guidance; final approval of the final version of the manuscript.

Samira Bührer-Sékula: Writing of the manuscript; analysis and interpretation; effective participation in the research guidance; intellectual participation in the propaedeutic and/or therapeutic conduct of the studied cases; final approval of the final version of the manuscript.

Mariane Martins de Araujo Stefani: Study concept and design; data collection, analysis and interpretation of data; statistical analysis; writing of the manuscript; data collection, analysis and interpretation; effective participation in the research guidance; intellectual participation in the propaedeutic and/or therapeutic conduct of the studied cases; critical review of the literature; final approval of the final version of the manuscript.

Maria Lucia Fernandes Penna: Study concept and design; data collection, analysis and interpretation of data; statistical analysis; writing of the manuscript; data collection, analysis and interpretation; effective participation in the research guidance; intellectual participation in the propaedeutic and/or therapeutic conduct of the studied cases; critical review of the literature; final approval of the final version of the manuscript.

## Conflicts of interest

The authors declare no conflicts of interest.
